# Sparsifying machine learning models identify stable subsets of predictive features for behavioral detection of autism

**DOI:** 10.1186/s13229-017-0180-6

**Published:** 2017-12-19

**Authors:** Sebastien Levy, Marlena Duda, Nick Haber, Dennis P. Wall

**Affiliations:** 1 Department of Pediatrics, Division of Systems Medicine, Stanford University, Stanford, CA USA; 2 Department of Biomedical Data Science, Stanford University, Stanford, CA USA; 3 Institute for Computational and Mathematical Engineering, Stanford University, Stanford, CA USA

**Keywords:** Autism, Autism spectrum disorder, ASD, Autism screening, Autism diagnosis, Machine learning, Sparse machine learning

## Abstract

**Background:**

Autism spectrum disorder (ASD) diagnosis can be delayed due in part to the time required for administration of standard exams, such as the Autism Diagnostic Observation Schedule (ADOS). Shorter and potentially mobilized approaches would help to alleviate bottlenecks in the healthcare system. Previous work using machine learning suggested that a subset of the behaviors measured by ADOS can achieve clinically acceptable levels of accuracy. Here we expand on this initial work to build sparse models that have higher potential to generalize to the clinical population.

**Methods:**

We assembled a collection of score sheets for two ADOS modules, one for children with phrased speech (Module 2; 1319 ASD cases, 70 controls) and the other for children with verbal fluency (Module 3; 2870 ASD cases, 273 controls). We used sparsity/parsimony enforcing regularization techniques in a nested cross validation grid search to select features for 17 unique supervised learning models, encoding missing values as additional indicator features. We augmented our feature sets with gender and age to train minimal and interpretable classifiers capable of robust detection of ASD from non-ASD.

**Results:**

By applying 17 unique supervised learning methods across 5 classification families tuned for sparse use of features and to be within 1 standard error of the optimal model, we find reduced sets of 10 and 5 features used in a majority of models. We tested the performance of the most interpretable of these sparse models, including Logistic Regression with L2 regularization or Linear SVM with L1 regularization. We obtained an area under the ROC curve of 0.95 for ADOS Module 3 and 0.93 for ADOS Module 2 with less than or equal to 10 features.

**Conclusions:**

The resulting models provide improved stability over previous machine learning efforts to minimize the time complexity of autism detection due to regularization and a small parameter space. These robustness techniques yield classifiers that are sparse, interpretable and that have potential to generalize to alternative modes of autism screening, diagnosis and monitoring, possibly including analysis of short home videos.

## Background

Autism spectrum disorder (ASD) is a developmental neuropsychiatric disorder characterized by impairments in social interaction, communication (both verbal and non-verbal), and restricted, repetitive behaviors. The most recent estimates by the Centers for Disease Control indicate that autism affects one in 68 children in the USA and is much more common in males, affecting as many as one in 42 boys as compared to one in 189 girls [1]. The average age of diagnosis for ASD is 4.5 years, though parents often identify developmental concerns within the child’s first year of life, even as early as 6 months of age [2], and clinicians report that reliable diagnoses can be made as early as age 2 [3].

Currently, the diagnosis of autism is based on behavioral symptoms alone. A typical diagnostic appointment consists of a multi-hour behavioral evaluation by a team of clinicians, usually in a specialized diagnostic clinic or developmental medicine center and only after referral from the child’s general pediatrician. During the diagnostic encounter, trained specialists will administer a battery of behavioral instruments and rating scales, which are standardized and can aid clinicians in reaching a best-estimate diagnosis. One of the most commonly utilized behavioral instruments is the Autism Diagnostic Observation Schedule (ADOS) [4], which is considered a gold-standard diagnostic tool. The ADOS is an observation-based clinical assessment that is broken into four modules based on age and language level: Module 1 is intended for young children with no or single-word speech, module 2 is intended for individuals with phrase speech, module 3 is intended for verbally fluent children, and module 4 is intended for verbally fluent adolescents and adults. The ADOS administrator will participate in a number of standardized activities with the child and subsequently answer a set of 28–30 questions about the child’s behavior during the activities. Each answer is coded on a scale from 0 to 3, with higher codes indicating more severe impairments in each measured behavior. Domain subscores and a total score are calculated by converting codes of 3 to 2 and totaling the codes from the appropriate subset of questions, and from these scores, a final classification of autism, autism spectrum, or not met is reached. In the original ADOS-G [5], subscores for communication, social interaction, and restricted, repetitive behavior (RRB) domains were calculated, but only the social and communication domains were used to determine the final classification. In the revised ADOS-2 [6] scoring algorithm, communication and social interaction were combined into a single social affect domain. The ADOS-2 also calculates a RRB domain, and both the social affect and RRB domains are used in determining the final classification. In addition to domain/total scores and a classification, the ADOS-2 also provides an algorithm for calculating a comparison score, which ranges from 1 to 10 and is meant to capture autism severity after calibration for age and language level. The total time for administration and scoring of the ADOS is approximately 60 min.

Due to the rigorous and time-consuming nature of diagnostic examinations for autism, many diagnostic centers have expanding waiting lists for appointments as the increasing demand exceeds their capacity to see patients. This bottleneck translates to delays in diagnosis of 13 months and longer for minority or lower socio-economic status groups. These delays can also delay insurance coverage and access to behavioral therapies [7, 8]. These issues indicate that there is a need for short, easily accessible, and accurate risk assessments for ASD both to provide feedback to parents and to provide clinics better abilities to triage and manage their patients. In our previous work, we applied machine learning approaches to identify minimal sets of behavioral features from commonly used behavioral instruments that yielded high accuracy in distinguishing children with ASD from those without ASD [9–12], as well as from children with other developmental delays [13]. In the present study, we focus on expanding the generalizabilty of those approaches, with specific focus on observation-based data from modules 2 and 3.

While identifying a minimally viable set of features for behavioral detection of varying forms of autism is an important step forward in an effort to construct a clinical process that reduces time to diagnosis, the models derived from our earlier work [12] did not account for instances when core features of the model could not be answered. As a consequence, these models may suffer from a lack of generalizability, such as in instances when the answer to one or more questions cannot be given, a phenomenon we might expect to be common in clinical practice. The present study attempts to address this limitation by focusing on creating more robust models that explicitly account for missing features. Specifically, we focus on data-driven identification of a small set of behavioral features on which several types of classifiers yield high accuracy to characterize an underlying structure in the data that is important under a variety of optimization criteria—that is, the same features used within and across models—and that confers an inherent robustness to the task of classification. The work here uses three guiding principals for the use of machine learning in building a process for faster autism detection, namely:

- To evaluate model accuracy, we computed the area under the receiver operating characteristic (ROC) curve. This metric deals well with label imbalance and remains agnostic to thresholding choices made for classification (i.e., tradeoff between false positive and false negative rates). Our objective was to reach accuracies close to those of the full ADOS test and when available to the best-estimate clinical diagnosis.
- A desired behavior for the selected models was simplicity or parsimony. A model with less parameters and more regularization (high penalization, small tree depth) will have better generalization, more stability to noisy data, and less over-fitting.
- Finally, a crucial part of our model selection was the potential for clinical application, and our ability to interpret exactly how the model’s features influence the detection of ASD. Interpretable models should be preferred. Linear models for regression, logistic regression, LDA, and linear SVM provide an intuitive interpretation on how much (value) and how (sign) each feature is used in the classification. Simple decision trees explicitly show how features are used together to decide if a patient has high risk for autism. With that knowledge, the selected model should result in a better understanding of the characteristics of ASD within each subject.

Using these guiding principles, we tested our approach on an aggregated collection of databases for ADOS modules 2 and 3. To derive sound estimates of the accuracies that could be reached on unseen data and avoid overfitting due to testing too many models, we selected only one or two models per module based on the three above criteria—accuracy, simplicity, and interpretability—and tested them on 20% of the data. We show the outcomes in light of the above criteria and describe the potential contribution of chosen models to the clinical detection of autism as well as their limitations. Finally, we discuss how the derivation of a quantitative phenotype for autism via the use of smaller sets of features in an interpretable machine learning model could help to accelerate the diagnostic process and therefore help ameliorate bottlenecks in access to care.

## Methods

### Data sample and preprocessing

We aggregated item-level Autism Diagnostic Observation Schedule (ADOS) module 2 and module 3 score sheets from four autism spectrum disorder (ASD) data repositories: the Boston Autism Consortium (AC), Autism Genetic Resource Exchange (AGRE), Simons Simplex Collection (SSC), and the Simons Variation in Individuals Project (SVIP). The module 2 data set consisted of 1389 subjects (1319 ASD, 70 non-ASD), and the module 3 data set consisted of 3143 subjects (2870 ASD, 273 non-ASD). Subjects were classified as ASD or non-ASD based on best-estimate clinical diagnosis where available, and for the small subset where clinical diagnosis was not available (*n*=75 module 2, *n*=125 module 3), the ADOS algorithm outcome was used to define the class. For module 3, the controls had a mean age of 109 months (std dev = 35 months) and children with ASD had a mean age of 115 months (std dev = 38 months; average 116 months (std dev = 38 months) for classic autism and 114 (std dev = 38 months) for autism spectrum). For module 2, controls had a mean age of 60 months (std dev = 28) and children with ASD had a mean age of 83 months (std dev = 38; 85 (std dev = 38) for classic autism and 74 (std dev = 36) for autism spectrum). For module 3, our non-ASD control set consisted of 137 males and 136 females and our ASD cohort consists of 2420 males and 450 females. For module 2, our non-ASD control set consisted of 41 males and 29 females, and our ASD set consists of 1060 males and 259 females.

Before beginning our machine learning pipeline, we performed preprocessing steps on our dataset. Both modules 2 and 3 contained missing value codes (e.g., answer codes “8,” “9,” “N/A”). Module 2 feature A3 (speech abnormality) was missing 2.3% of the time, with all other features missing answers in less than 1% of the subjects. Module 3 had more missing values, with 1.8% of feature A3 (echolalia) missing, 78.6% of feature B3 (vocalized nonverbal communication) missing, and 8.2% of D3 (self-injury) missing. We elected to incorporate these missing values as features for model development. First, for each item in modules 2 and 3, we created a binary “not answered” feature that captured whether or not the administrator answered the question, irrespective of the code provided. We grouped answers that indicated “N/A” (e.g., an 8 or 9) together with answers that were omitted and left blank. We then coded this binary feature (1 if X is missing and 0 if X is present) as X-missing, so, for instance, if feature A3 was coded as missing, A3-missing took the value 1. Although this doubled our initial feature set, it allowed us to interpret how the inability to answer a question, or inability to assess a certain behavior, related to diagnosis in both modules. In addition to the primary items from the instruments and the missing features, we included age and gender as features, resulting in a 58-item feature set for both module 2 and module 3. Next, we performed a normalization step to guarantee that our feature data were all on a uniform scale. Since all of the original ADOS items range from 0 to 3, we transformed the rest of the features to be on the same scale. Gender and other binary indicators were represented by 0’s and 3’s, and age was rescaled to fit in the 0–3 range.

We used the best-estimate clinical diagnosis or ADOS classification when diagnosis was not available as the prediction class for our machine learning experiment. When using regression, to increase the granularity of our prediction class, we split our subjects into three diagnostic groups at training time: autism, spectrum, and non-ASD. The autism class included diagnoses of autism, autistic disorder, and classic autism, while the spectrum class included diagnoses of autism spectrum disorder/ASD, Asperger’s, high-functioning autism, and pervasive developmental disorder-not otherwise defined (PDD-NOS). In our machine learning analyses, these group labels would then be converted into integers (non-ASD (0), spectrum (1), and autism (2)) which captured the increasing severity of the classes. This assignment applies only to the linear regression tasks but is reasonable in order to test whether increased ordinal structure on a regression task leads to different performance on a two-class (0 vs 1 and 2) classification.

### Machine learning

We tested the performance of 17 unique machine learning classifiers on both our module 2 and module 3 feature sets to accurately predict the diagnosis of ASD or non-ASD, using a small stable subset of features yielding comparable accuracy to the complete feature set. Training and testing of our models was performed in Python using the package scikit-learn [14].

We chose to test models from five distinct classifier families: linear regressions (thresholded for classification), nearest neighbor models, general linear models (classifiers, as opposed to regressors), support vector machines, and tree-based methods. Table 1 contains a summary of the different models we tested. Some classifier families provide a built-in sparsifying parameter—for example, Lasso penalizes the weights using the *L*_1_ norm. By increasing this penalty term, one can force the model to use fewer features to generate predictions [15]. However, some of our models, namely, most of the kernels in support vector machines (SVMs) and tree-based classifiers, do not have an easily tunable sparsifying parameter. For these classifiers, we added a feature selection technique to the training phase. Three techniques for scoring each feature individually were used, detailed in Table 2: ANOVA, nonzero coefficients of a Lasso trained on the data or more important features of a full decision tree trained on the data (referred to as tree in subsequent sections). The number of features selected was tuned using a parameter as detailed in Table 2. This parameter was then treated as the sparsifying coefficient for the sparsified model (feature selection followed by classification performed on selected features). We annotated this sparsified model with the feature selection method (“ANOVA-,” “Lasso-,” or “Tree-”; so, for instance, a SVM trained on a subset of features found to be important by application of Lasso was written “Lasso-SVM”) as a prefix. For comparison, we also used the base model without any additional feature selection, denoted with the prefix “NS-.” In this case, and for the second phase, grid search was used to optimize non-sparsifying regularization parameters (e.g., *L*_2_ regularization coefficient) only.

**Table 1:** Summary of tested classifiers

Classification family	Models used	Built-in sparsifying coefficient, other penalization	Under-sampling used	Relevance
Penalized linear regression	Linear Regression	*L*_1_ penalization	Yes	∙ Very interpretable
	Lasso	*L*_2_ penalization		∙ Simple model
	Ridge			∙ Linear like ADOS
	Elastic net			∙ Can use gradation in label
	Relaxed Lasso			(ASD vs spectrum)
Nearest neighbors	Nearest shrunken centroids	*L*_1_ penalization	Yes	∙ Can identify subgroups within classes,
				which is likely for our sample
				∙ Simple model
General linear models for classification	LDA (*L*_1_)	*L*_1_ penalization	No	∙ Simple model
	Logistic regression (*L*_1_, *L*_2_)	*L*_2_ penalization		∙ Interpretable
				∙ Based on linear assumptions
Support vector machines	Linear kernel (*L*_1_)	*L*_1_ penalization	No	∙ Can capture more complex shapes in data when using nonlinear kernels
	Polynomial kernel	Regularization parameter		
	Radial kernel			
	Exponential kernel			
Tree-based classifiers	Decision tree	Tree depth	No	∙ Performs well on categorical data
	Random forest	Number of trees		∙ Better captures feature interactions
	Gradient boosting			∙ Tree is interpretable
	AdaBoost			∙ Boosting techniques often gives higher accuracy than simpler models

We trained and tested 17 unique machine learning classifiers on both our module 2 and module 3 training data sets. Linear regressions models were trained to differentiate autism, spectrum, and non-ASD (3 prediction classes) but tested to detect only ASD from non-ASD

**Table 2:** Summary of feature selection techniques used for classifiers without sparsity enforcing parameters

Feature score	Description	Sparsifying coefficient	Advantages
ANOVA	The *k* most discriminative features	*k*	∙ Simple test
	when doing the ANOVA test		∙ Fast
			∙ A priori information on what features
			would not be useful in classification using
			only the variance for each features
Lasso	Nonzero coefficients of the Lasso	*L*_1_ coefficient	∙ Linear model
	trained on the data for a given *L*_1_ coef		∙ Features used by a more parsimonious model
Tree	The *k* most important features when building	*k*	∙ Good with categorical data as it can use
	a full decision tree on the data		multiple cuts per feature, unlike linear models

The third column gives the parameter that will be used by the full model as the sparsifying coefficient for the grid search

### Feature reduction

The first phase of our machine learning pipeline consisted of identifying a reduced feature set that was subsequently used to build our final models. First, we randomly split our preprocessed data into distinct training (80%) and testing (20%) sets, with the same proportion of ASD to non-ASD subjects in each set. Preserving a portion of our data strictly for testing enabled us to choose our final model based on how well it could generalize to unseen data, preventing the selection of a model overfit to our training set.

To identify an optimal subset of features for each of our models, we performed a stratified 10-fold cross-validation (feature selection CV) with a nested grid search on each fold, using only our training data set. We corrected imbalance in class size by setting class weights inversely proportional to the class sizes. When classifiers/classifier implementations did not allow for different class weights, we used undersampling in each fold of the feature selection CV, resulting in a 1:1 ratio of ASD to control data in each fold. The grid search technique searches for the set of parameters that optimizes the performance of the model, traditionally measured by classification accuracy. For our purposes, we altered the traditional grid search method in two ways. First, due to our class imbalance, we utilized the area under the receiver operating characteristic (AUC_ROC_) and area under the precision-recall curve (AUC_PR_) as our performance metrics instead of basic accuracy. Second, we added a penalization term to the grid search objective function to enforce sparsity in the model. To do this, we found the set of parameters *θ* that maximize the penalized objective of the equation below where AUC denotes the area under curve of either ROC or PRC.

$$\begin{aligned} \hat \theta &= \text{argmax}_{\theta} \left(M_{{sparsity}}(\theta)\right) \\ &= \text{argmax}_{\theta} \left(\! {AUC}(\theta) - \mu \frac{\text{Number of features used}(\theta)}{\text{total number of features}}\right) \end{aligned} $$

By penalizing the use of more features, we force some bias in the selected algorithm but decrease the variance by decreasing the model complexity. This approach can be seen as similar to Lasso but for the *L*_0_ norm (how many features are in our model) and will be called *L*_0_ regularization in the following sections. This grid search metric improves the selection of simpler, sparser models that highlight important features and limit overfitting. After testing different values on a first cross-validation, *μ* = 0.1 Gr was found to be a good coefficient for the regularization as it yielded a small number of features while remaining within 1% accuracy of the non-regularized version.

In addition to the above, and to further enforce sparsity, we used the one standard error rule. We chose the most parsimonious model whose error is no more than one standard error above the error of the best model [16]. In our case, we defined parsimony as the amount of regularization, i.e., *L*_1_ or *L*_2_ penalization, small tree depth, and/or small numbers of trees. We computed the average objective function on all folds, selected the classifier with the maximum value, computed its standard error on all folds, and selected the most parsimonious (regularized) parameters within one standard error of that highest objective. This corresponds to the maximization problem over parsimony in the equation below, where we define $\hat \theta $ to be the optimal set of parameters without the one standard error rule and *SE* to be the standard error.

$$\begin{aligned} \hat \theta_{1se} &= \text{argmax}_{\theta} \,\,\texttt{parsimony}(\theta) \\ & \text{s.t.}\,\, M_{{sparsity}}(\theta)\geq M_{{sparsity}}(\hat \theta) - SE\left(M_{{sparsity}}(\hat \theta)\right) \end{aligned} $$

**Fig. 1:**
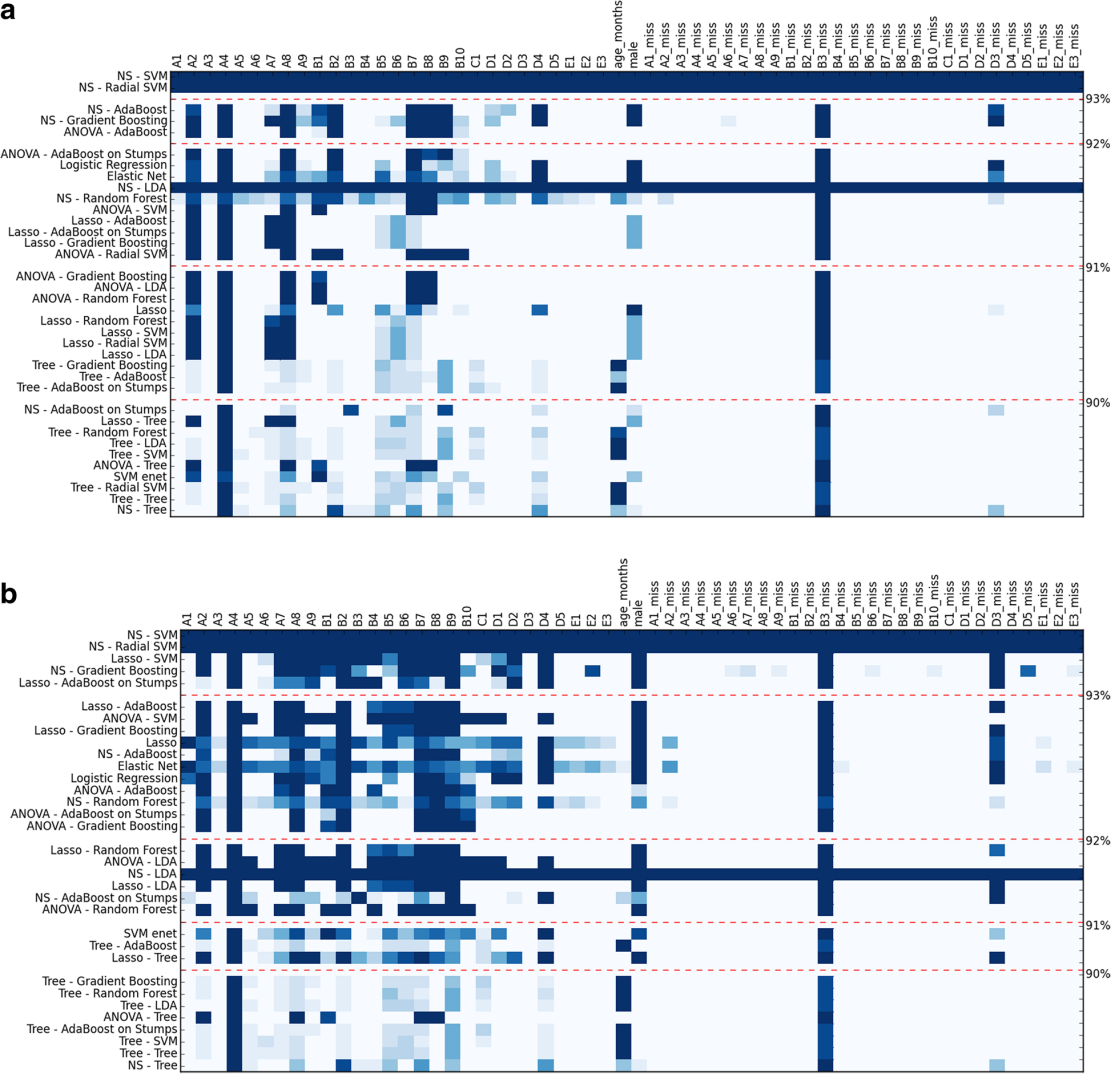
Heatmap of features used on the different folds for module 3. The darker the color of the cell, the more the feature was used in the different folds of the feature selection cross-validation (CV). Classifiers were sorted along the *y*-axis such that those with the highest AUC_ROC_ function were at the top. Color intensity of each cell denotes how often that feature was selected in all folds of the feature selection CV for that model. The top figure (**a**) used *L*_0_ regularization, and the bottom one (**b**) did not. Both used the one standard error rule

Once this grid search was performed, we constructed a heatmap to determine how often each of our features were selected among our models. This heatmap is a table where each cell corresponds to a feature and a classifier (see Figs. 1 and 6). Using this heatmap, we compared the feature sets among the best performing models to arrive at a reduced feature set that is stable both within and between classifiers. It can also be used to test our assumption that the same subset of features will be selected by multiple classifiers to reach optimal accuracy. In the experiments, we computed two heatmaps, using the one standard error rule and *L*_0_ regularization for the first, and only the one standard error rule for the second. We then chose sets of 5 and 10 most used features with the *L*_0_ penalization heatmap. When two features were used equally, we broke the ties by choosing the most frequently utilized in the non-regularized heatmap. We labeled these feature sets as reduced-5 and reduced-10, respectively.

### Model training and testing

The second phase of our machine learning pipeline consisted of training each of our models using the reduced feature set identified in phase 1. To train our models, we used another stratified 10-fold cross-validation approach with nested grid search (model tuning CV) in the same way as the feature selection CV, but in this phase, the objective function was set to find the values for model hyper-parameters (such as *L*_2_ norm or tree depth) that maximize only the *AUC*_*ROC*_ without any additional sparsity-enforcing parameter.

For each model, we selected the hyper-parameters that gave the best overall score over all folds, and, using these, we estimated which classifier should perform well by averaging the corresponding *AUC*_*ROC*_ for each fold of our model tuning CV. In addition to this performance estimation, we took into account two other objectives when choosing our final model: simplicity and interpretability. For our purposes, simplicity is important because simpler and more parsimonious models generally decrease the chances of overfitting on the training data. Finally, we wanted to choose an interpretable model to gain more insight on how to reliably detect ASD. In practice, in this second phase, we selected all the models with high accuracy (close to ADOS accuracy) and selected the best trade-off between accuracy, simplicity, and interpretability from the remaining models. As an additional metric to distinguish models, we use the area under the PR curves (both AUCPRcontrol, counting controls as the positive class, and AUCPRASD, counting the ASD cohort as positive class).

The third and last phase of our pipeline was testing our final selected model from phase 2 on our dedicated testing set (20% of our original data) to see how well this model could separate ASD from non-ASD in completely unseen data. We computed the area under the three curves described previously to estimate how our selected model would perform on unseen data.

## Results

### ADOS module 3 (children with medium to low levels of autism symptom severity)

#### Reduced feature set

Using module 3, we obtained two heatmaps using the one standard error rule with and without *L*_0_ regularization that can be found in Fig. 1. The full grid search results can be found in Table 3. Taken together, these heatmaps suggested that the reduced feature set was stable, as the same features were highlighted by both.

**Table 3:** Grid search results of the classifiers for module 3, with and without *L*_0_ penalization

Classifier	Linear reg	Lasso	Ridge	Elastic net	Relaxed Lasso	L1 logreg	L2 logreg	LDA
*L*_0_ penalized ROC AUC	84.8	89.1	84.9	89.5	90.2	90.0	82.5	89.4
Associated real ROC AUC	92.4	90.6	92.4	91.7	91.7	91.8	92.2	90.6
Features used with *L*_0_	*44.1*	8.4	*43.2*	12.9	8.6	10.7	*56.5*	7.0
Not penalized ROC AUC	92.3	92.7	92.5	92.6	92.5	92.8	92.2	91.9
Features used without *L*_0_	*43.9*	23.3	*40.4*	25.8	23.4	19.4	*56.5*	20.0
Classifier	pSVM	rSVM	eSVM	L1 lSVM	Grad Boost	AdaBoost	Rand Forest	Tree
*L*_0_ penalized ROC AUC	88.9	89.3	*49.3*	89.5	90.0	90.7	89.9	88.5
Associated real ROC AUC	90.1	91.1	*50*	91.1	91.1	92.1	90.6	89.6
Features used with *L*_0_	6.4	10.0	4.0	9.4	6.4	8.3	7.0	6.4
Not penalized ROC AUC	91.6	93.2	*50.0*	92.8	93.1	93.1	91.9	90.0
Features used without *L*_0_	13.6	*58.0*	*58.0*	*38.1*	20.7	14.5	13.2	16.2

pSVM, rSVM, eSVM, and lSVM correspond to different kernels for SVM (polynomial, radial, exponential, and linear) and logreg to logistic regression. Italicized data points highlight the worst performing models (too many features used and/or poor performance)

The *L*_0_ regularization heatmap highlighted A2-, A4-, A8-, and B3-missing as the top features, and when considering the non-regularized heatmap, B2 was also frequently chosen. We denoted this collection of behavioral elements as our reduced-5 feature set. For the reduced-10 feature set, we added B7, B8 by the *L*_0_ regularized heatmap and D4, gender and D3-missing from the heatmap without regularization. Finally, B9 was also frequently used in the first heatmap and A7, B1, B6, and D2 on the second one. The categories of the chosen feature are summarized in Table 4.

**Table 4:** Summarized description of the features chosen by the feature reduction process for module 3

Feature	Category
A2	Language (intonation, volume, rhythm)
A4	Language (words/phrases)
A8	Language (conversation)
B3-missing	Language: examiner could not test nonverbal communication linked to language production
B2	Social interaction (facial expressions)
B7	Social interaction (social overtures)
B8	Social interaction (social overtures, attention)
D4	Behavior (repetitive behavior)
D3-missing	Behavior: examiner could not test self injurious behavior
Gender	Gender of the patient

#### Selected classifier

##### Reduced-10 feature set

**Fig. 2:**
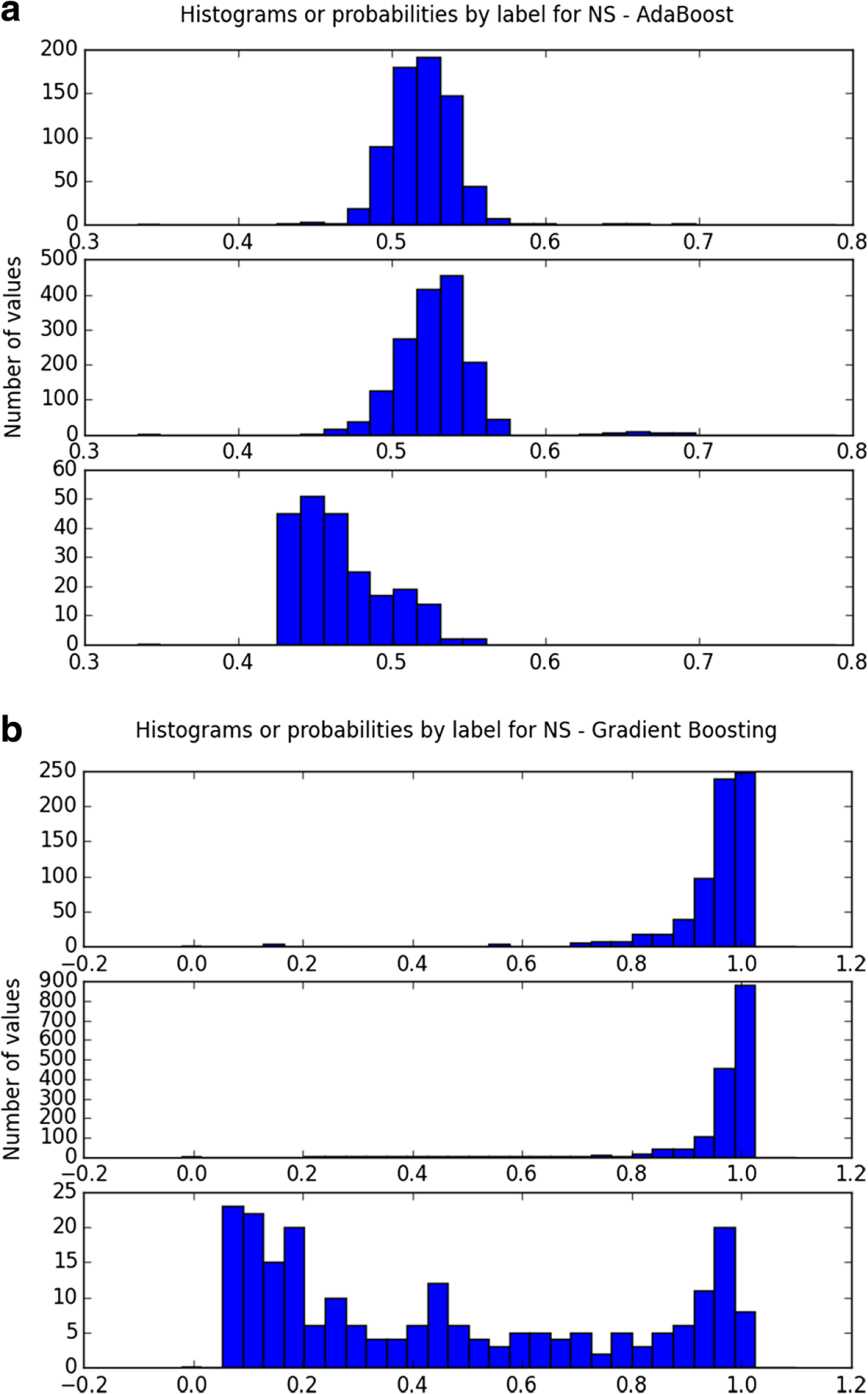
Histogram of predicted probabilities of the two discarded models on the cross-validation set. The *x*-axis denotes the predicted probabilities and the *y*-axis the number of subjects. The top figure (**a**) corresponds to AdaBoost and the bottom figure (**b**) to Gradient Boosting. AdaBoost probability plot is unstable, and gradient boosting has a high rate of misclassification among the control subjects

Four classifiers performed better on the reduced-10 feature than the others: SVM, boosting on decision trees (with both AdaBoost and Gradient Boosting methods) and logistic regression with *AUC*_*ROC*_ of all three around 0.93. However, when considering our three criteria for choosing the best model, logistic regression and linear SVM were simpler and more interpretable than the the boosting methods for similar performance. Although logistic regression tends to be more interpretable, we provide results for both classifiers. Moreover, it supported the ADOS rating criterion, suggesting that the class distinction had an underlying linear structure. By looking at the probability plots for these three well-performing classifiers (see Fig. 2), we can see that the AdaBoost probability plot was unstable and SVM and gradient boosting yielded a higher rate of misclassification among controls than the logistic regression model. In the performed grid search, the optimal regularization coefficient (inverse of the penalization coefficient) was C = 1 and *L*_2_ penalization performed slightly better than *L*_1_ penalization.

**Fig. 3:**
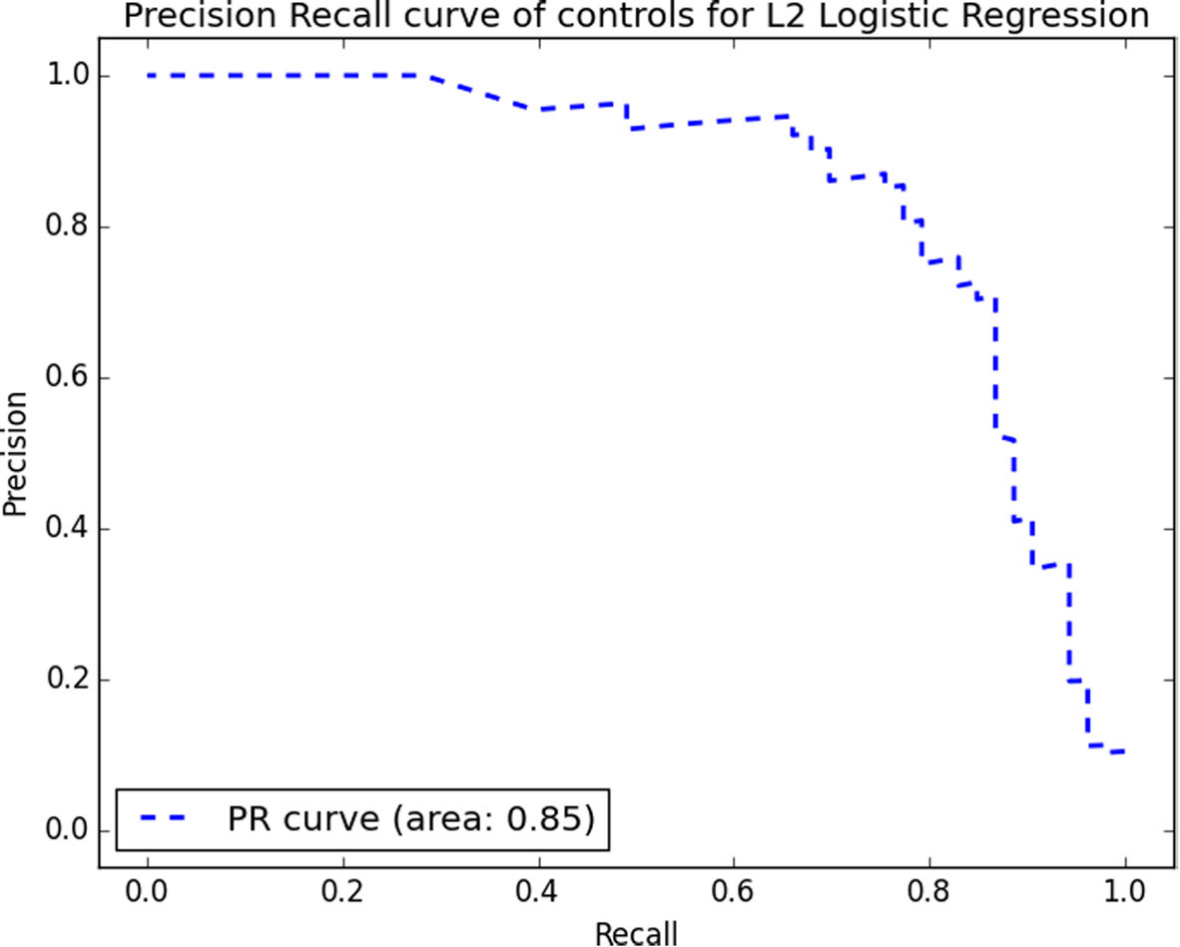
Precision-recall curve of controls for logistic regression with *L*_2_ penalization on the test set. Above 0.8 of recall, precision decreases drastically. Precision-recall curve of ASD is the whole square (area under the curve is ∼ 1)

**Fig. 4:**
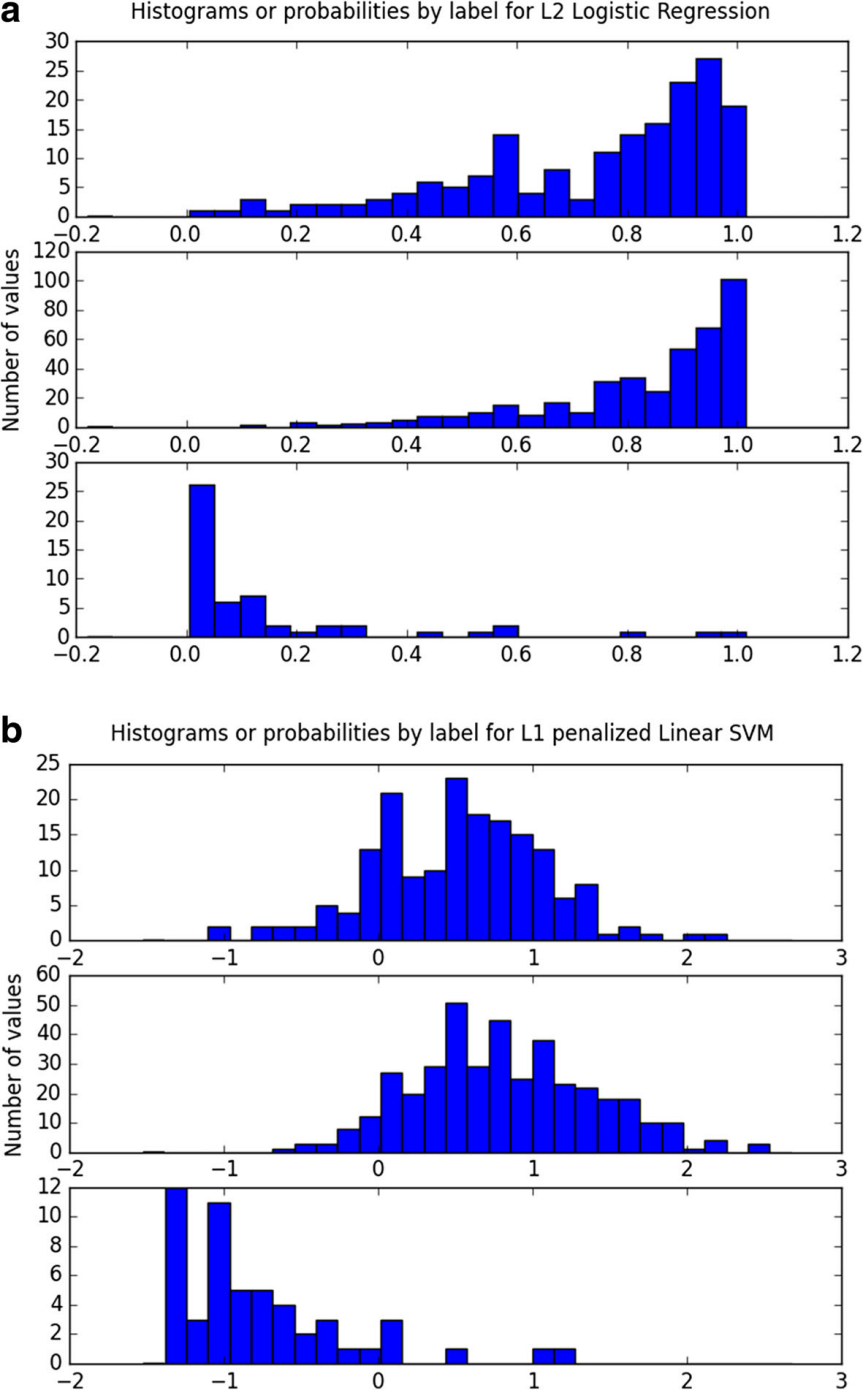
Histogram of predicted probabilities for logistic regression with *L*_2_ penalization (**a**) and linear SVM with *L*_1_ penalization (**b**) on the test set. The *x*-axis denotes the predicted probabilities for logistic regression and the decision function for SVM and the *y*-axis represents the number of subjects. Each subplot correspond to a different label

Lastly, we computed the performance of our final logistic regression and SVM models on the test set, composed of 20% of our original data. Logistic regression, on the reduced-10 feature set, achieved *AUC*_*ROC*_ = 0.95 and a balanced accuracy (*Bal*_*Acc*_) of 0.90, while SVM performed lower with *AUC*_*ROC*_ = 0.95 and *Bal*_*Acc*_ = 0.80. Other statistics can be found in Table 5. AUC for precision-recall indicated that it is harder to detect controls than it is to detect children with ASD, an expected phenomenon given the compositional biases of our dataset. The full PRC curve on Fig. 3 indicated that for a recall above 0.8, we could not achieve a reasonable precision. Figure 4 shows that most of the controls were well classified and most of the ASD cases were identified as well. The cutoff area inducing reasonable accuracy is quite large (between 0.2 and 0.4), suggesting that we have a stable prediction. Classic autism subjects, i.e., the higher severity group, were more difficult to classify than the spectrum. This is counter-intuitive—one would expect them to be more distinguishable. We defer to the “Discussion” section reasoning as to why this might be the case.

**Table 5:** Summary of accuracies for modules 2 and 3 with best classifier, best parameters, and different feature sets

Module	3	3	3	2	2	2
Number of features	10	10	5	5	5	5
Best classifier	L2 LR	L1 Lin SVM	L2 LR	LDA	L1 Lin SVM	L2 LR
Optimal parameters	*C* = 1	*C* = 0.5	*C* = 10	*S* = 0.8	*C* = 0.5	*C* = 0.05
Area under ROC	0.95	0.95	0.93	0.93	0.93	0.92
Precision	0.99	0.99	0.99	0.98	0.98	0.98
Recall/sensitivity	0.90	0.95	0.88	0.97	0.98	0.93
Specificity	0.89	0.87	0.89	0.50	0.58	0.67
Balanced accuracy	0.90	0.90	0.88	0.74	0.78	0.80
F1 score	0.94	0.97	0.93	0.97	0.98	0.95

LR denotes logistic regression, L1 Lin SVM denotes *L*_1_ penalized linear SVM, and S denotes the LDA shrinkage parameter

Given the imbalance in the gender ratio found in the non-ASD and ASD data, we elected to perform the same test on classifiers trained on the top 9 features, excluding gender as a feature. Results were essentially unchanged; see Table 6 for details. Further, see Table 7 for correlations between age, gender, and chosen features. All correlations with age and gender are relatively low and therefore not likely to be impacting the classification.

**Table 6:** Summary of accuracies for module 3 with best classifiers and parameters for our 10-feature set, this time trained without gender

Module	3	3
Number of features	9	9
Best classifier	L2 LR	L1 Lin SVM
Optimal parameters	*C* = 1	*C* = 0.5
Area under ROC	0.95	0.95
Precision	0.99	0.99
Recall/sensitivity	0.89	0.95
Specificity	0.90	0.87
Balanced accuracy	0.90	0.91
F1 score	0.94	0.97

LR denotes logistic regression, and L1 Lin SVM denotes *L*_1_-penalized linear SVM

**Table 7:** Correlations between used features and age and gender for module 3

Feature	A2	A4	A8	B2	B7	B8	D4	Age_months	Male	ASD	B3_miss	D3_miss
A2	1.000	0.391	0.307	0.295	0.369	0.346	0.220	0.161	0.112	0.343	0.295	− 0.096
A4	0.391	1.000	0.214	0.149	0.274	0.317	0.306	0.024	0.150	0.309	0.220	0.017
A8	0.307	0.214	1.000	0.332	0.468	0.496	0.161	0.006	0.102	0.348	0.304	− 0.041
B2	0.295	0.149	0.332	1.000	0.367	0.328	0.084	0.133	0.106	0.315	0.354	− 0.023
B7	0.369	0.274	0.468	0.367	1.000	0.464	0.195	0.090	0.113	0.371	0.318	− 0.053
B8	0.346	0.317	0.496	0.328	0.464	1.000	0.216	0.008	0.103	0.350	0.281	− 0.041
D4	0.220	0.306	0.161	0.084	0.195	0.216	1.000	− 0.006	0.155	0.172	0.157	− 0.246
Age_months	0.161	0.024	0.006	0.133	0.090	0.008	− 0.006	1.000	0.010	0.054	0.070	− 0.061
Gender	0.112	0.150	0.102	0.106	0.113	0.103	0.155	0.010	1.000	0.164	0.157	0.016
ASD	0.343	0.309	0.348	0.315	0.371	0.350	0.172	0.054	0.164	1.000	0.338	0.090
B3_miss	0.295	0.220	0.304	0.354	0.318	0.281	0.157	0.070	0.157	0.338	1.000	− 0.075
D3_miss	− 0.096	0.017	− 0.041	− 0.023	− 0.053	− 0.041	− 0.246	− 0.061	0.016	0.090	− 0.075	1.000

##### Reduced-5 feature set

Using only the reduced-5 feature set, the grid search results suggested that AdaBoost, logistic regression, and gradient boosting performed best, with *AUC*_*ROC*_ close to 0.92. Following the same logic used for the reduced-10 feature set, we chose logistic regression with *L*_2_ penalization for our final model. Its optimal sparsity coefficient was *C* = 10, confirming the intuition that less regularization was necessary with fewer features.

**Fig. 5:**
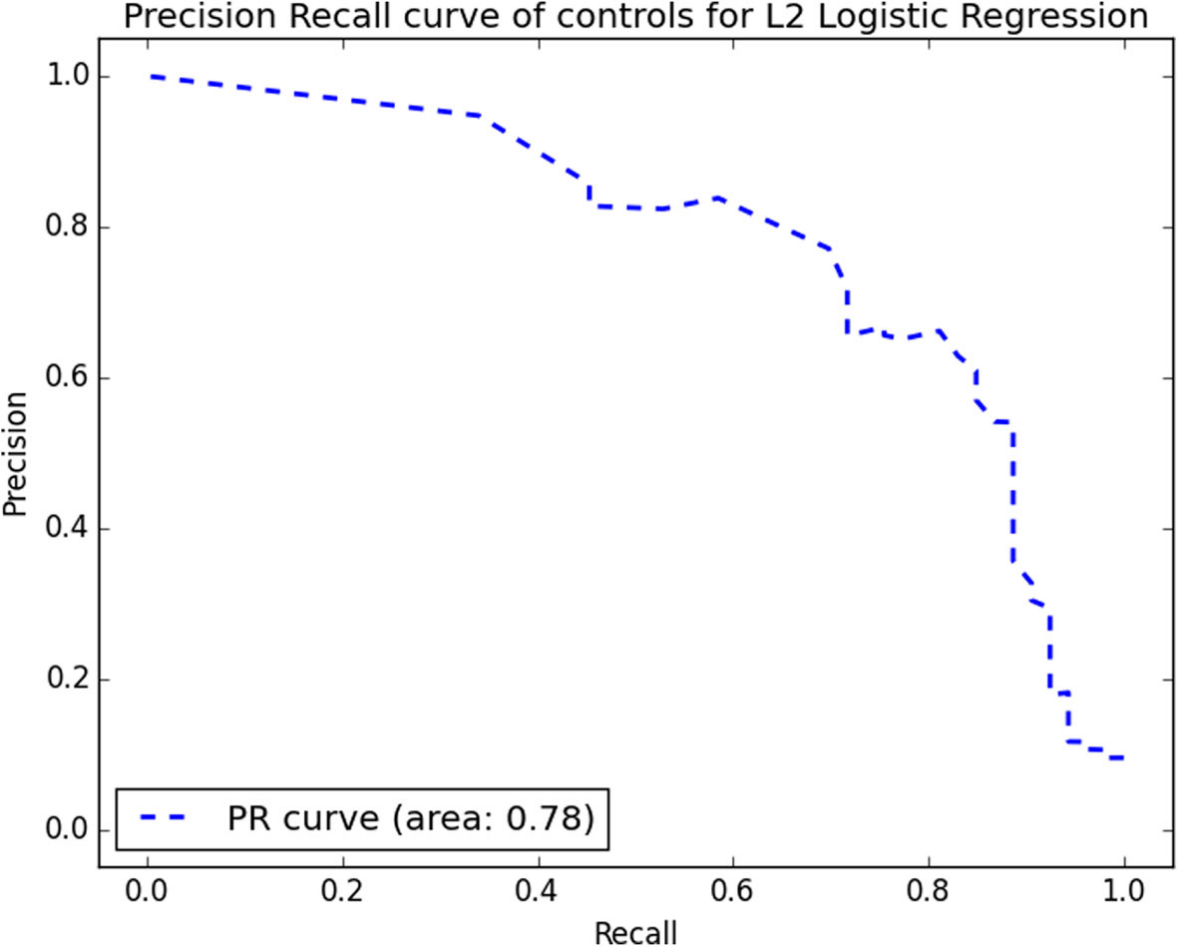
Precision-recall curve of controls for logistic regression with *L*_2_ penalization on the test set with only five features. Above 0.4 of recall, we reach 0.8 of precision. After 0.7, the precision decreases drastically

The final performance estimate yielded *AUC*_*ROC*_=0.93 and *Bal*_*Acc*_=0.88. Other statistics can be found in Table 5. Figure 5 shows that even though the *AUC*_*ROC*_ did not decrease much, achieving good precision and high recall was not possible.

### Module 2 (children with phrase speech)

#### Reduced feature set

**Fig. 6:**
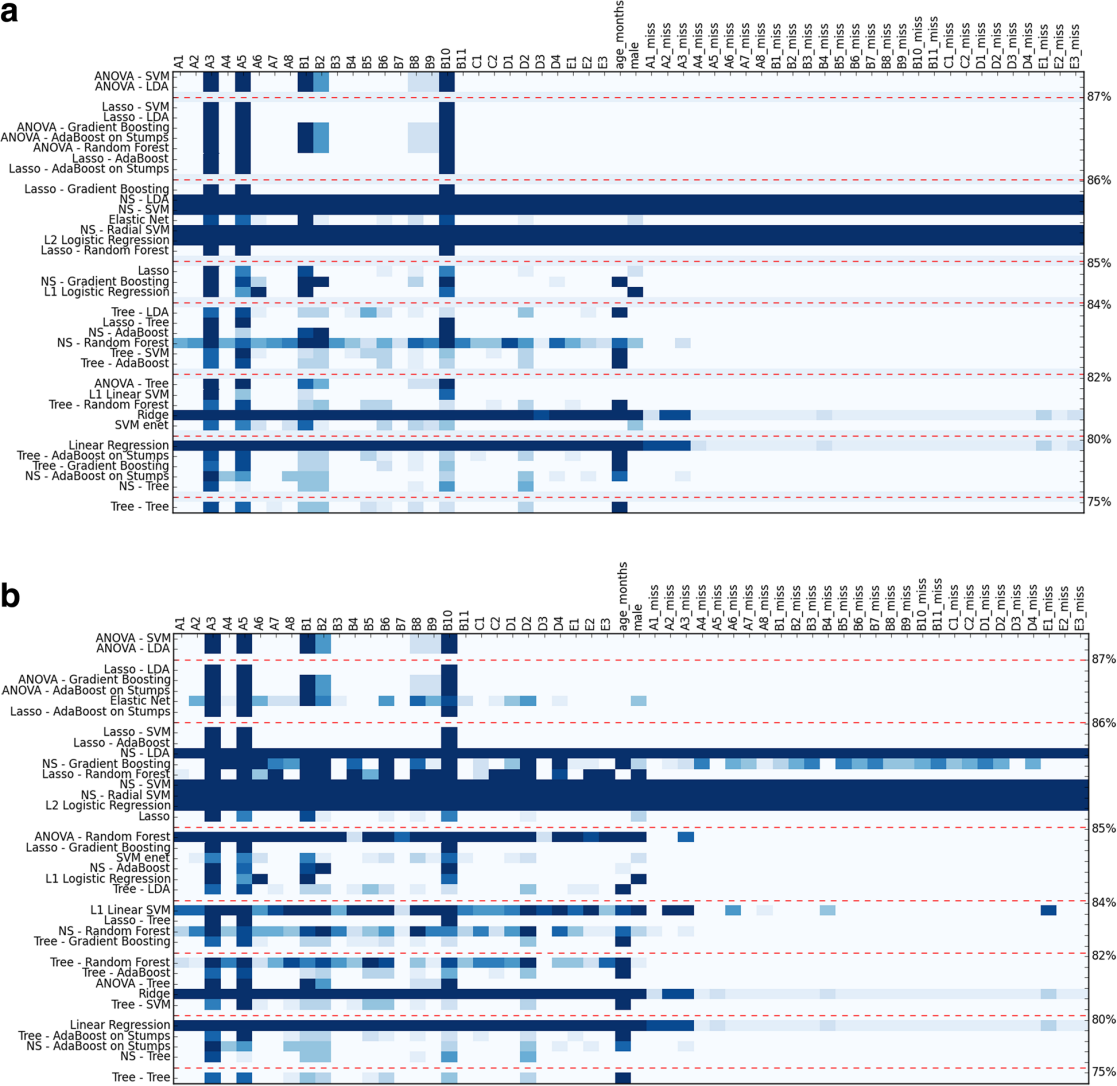
Heatmap of features used on the different folds for module 2. The darker the color of the cell, the more the feature was used in the different folds of the feature selection CV. Classifiers were sorted along the *y*-axis such that those with the highest AUC_ROC_ function appear at the top. Color intensity of each cell denotes how often that feature was selected in all folds of the feature selection CV for that model. The top figure (**a**) shows results with *L*_0_ regularization, and the bottom figure (**b**) shows results without regularization. In both analyses, we used the one standard error rule

Our ability to build a stable classifier was limited in part by the small size of the dataset available for module 2. We derived two heatmaps for module 2 using the one standard error rule, both with or without *L*_0_ regularization (Fig. 6). The full grid search results can be found in Table 8.

**Table 8:** Grid search results of the classifiers for module 2, with and without *L*_0_ penalization

Classifier	Linear reg	Lasso	Ridge	Elastic net	Relaxed Lasso	L1 logreg	L2 logreg	LDA
*L*_0_ penalized ROC AUC	73.7	84.1	75.4	84.6	84.3	83.3	75.4	86.5
Associated real ROC AUC	80.0	84.9	81.5	85.5	85.1	84.2	85.4	87.4
Features used with *L*_0_	*36.*5	4.4	*35.7*	5.3	4.4	5.4	*58.0*	5.0
Not penalized ROC AUC	79.3	85.1	81.2	86.3	85.3	85.9	85.3	87.4
Features used without *L*_0_	*35.4*	4.5	*34.*6	10.7	4.5	4.0	*58.0*	5.0
Classifier	pSVM	rSVM	eSVM	L1 lSVM	Grad Boost	AdaBoost	Rand Forest	Tree
*L*_0_ penalized ROC AUC	86.8	86.1	*49.3*	81.2	85.7	85.7	86.3	82.4
Associated real ROC AUC	87.7	86.6	*0.50*	81.6	86.6	86.6	86.2	83.0
Features used with *L*_0_	5.0	5.0	4.0	2.4	5.0	5.0	5.0	3.0
Not penalized ROC AUC	87.6	85.9	*50.0*	83.0	86.6	86.6	88.4	83.0
Features used without *L*_0_	5.0	12.0	4.0	*29.4*	5.0	5.0	*28.9*	3.0

pSVM, rSVM, eSVM, and lSVM correspond to different kernels for SVM (polynomial, radial, exponential, and linear) and logreg to logistic regression. Italicized data points highlight the worst performing models (too many features used and/or poor performance)

The *L*_0_ regularized heatmap shows A3, A5, B1, B2, and B10 to be the most highly selected features, which we denote as the module 2 reduced-5 feature set. The non-regularized heatmap confirmed these features to be top-ranked. Although age was selected quite often, we decided not to add it to the larger feature set because of the arbitrary age difference between the two classes in our dataset. No other feature was selected consistently, suggesting that five of the 29 total features captured within module 2 may be sufficient to identify autism from non-autism subjects. The categories and descriptions of chosen features are summarized in Table 9.

**Table 9:** Summarized description of the features chosen by the feature reduction process for module 2

Feature	Category
A3	Language (echolalia)
A5	Language (conversation)
B1	Social interaction (amount of maladjusted eye contact)
B2	Social interaction (facial expressions)
B10	Social interaction (social responses)

#### Selected classifier

The best performing classifiers on the reduced-5 feature set for module 2 were SVM, LDA, and logistic regression with *AUC*_*ROC*_ almost reaching 0.88. Each of the three models were simple and interpretable. Although polynomial kernel performed slightly better, for parsimony reasons, an SVM with a linear kernel and *L*_1_ penalization proved to be optimal. For all classifiers, ${AUC}_{{PR_{controls}}}~=~0.5$ and ${AUC}_{{PR_{ASD}}}~>~0.99$. Considering their similar performance and interpretability, we elected to test all of these models on the test set.

**Fig. 7:**
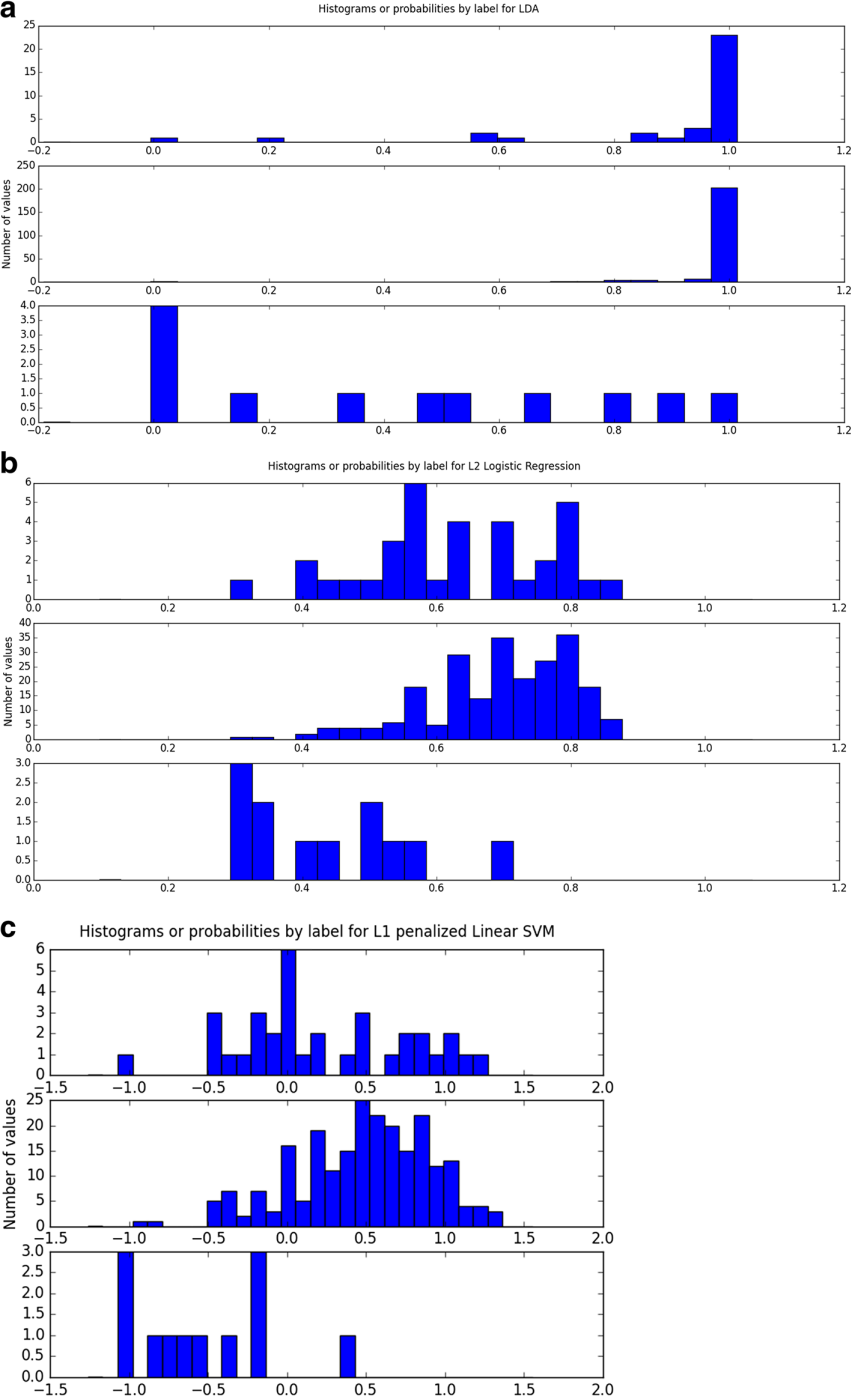
Histogram of predicted probabilities for LDA (**a**), logistic regression with *L*_2_ penalization (**b**), and linear SVM with *L*_1_ penalization (**c**) on the test set. The *x*-axis denotes the predicted probabilities (or decision function for SVM), and the *y*-axis shows the number of subjects. Each subplot corresponds to a different label

On the full training set (all subjects excluding the 20% held out from the test set), the optimal logistic regression model found in the grid search used *L*_2_ penalization and *C*=0.05, the optimal LDA model used shrinkage = 0.8, and the optimal SVM *C*=0.5. On the final test set, the LDA model exhibited *AUC*_*ROC*_=0.93, *Bal*_*Acc*_=0.74, and the SVM 0.93 and 0.80 and the logistic regression model 0.92 and 0.78, respectively. All statistics can be found in Table 5. Figure 7 shows the distribution of predicted probabilities from LDA, SVM, and logistic regression models. The small number of controls in the data set decreased the accuracy of the curve. We found high recall on detecting ASD using SVM and higher specificity using logistic regression. However, the ability of these classifiers to generalize to new data may be limited.

As for module 3 (and perhaps more so in the case of gender for module 2), there is a concern that imbalance between classes with age and gender limits these results. We computed the correlation matrix between age, gender, and chosen features; please see Table 10.

**Table 10:** Correlations between used features and age and gender for module 2

Feature	A3	A5	B1	B2	B10	Age_months	Gender
A3	1.000	0.405	0.272	0.322	0.329	0.350	0.049
A5	0.405	1.000	0.229	0.257	0.313	0.227	0.024
B1	0.272	0.229	1.000	0.283	0.257	0.105	0.057
B2	0.322	0.257	0.283	1.000	0.426	0.258	0.032
B10	0.329	0.313	0.257	0.426	1.000	0.286	0.023
Age_months	0.350	0.227	0.105	0.258	0.286	1.000	0.025
Gender	0.049	0.024	0.057	0.032	0.023	0.025	1.000

## Discussion

In this study, we aimed to identify a core subset of behavioral features from the gold-standard ADOS examination that can reliably discriminate between ASD and non-ASD cases. By considering the implication of missing answers as well as employing robust sparsity-enforcing feature selection methods during the first phase of our machine learning pipeline, we arrived at a novel subset of features from modules 2 and 3 that consistently optimized performance both within and across classifiers. Once the reduced feature sets were identified for each module, we fit each of our models to the reduced feature data set without any sparsifying coefficients and evaluated their performance on our dedicated test set. When choosing our final model for each module, we considered simplicity and interpretability in addition to overall classification performance. We found that logistic regression with *L*_2_ regularization performed best on the module 3 (*AUC*_*ROC*_ = 0.93) and, depending on the task, logistic regression or SVM performed best on the module 2 (*AUC*_*ROC*_ = 0.92 and 0.93). The full results are reported in Table 5. These results not only indicate that there is a stable subset of questions that contains the necessary information to distinguish ASD from non-ASD cases, but also points to an underlying linearity in the ADOS, since most top-performing models are linear. Table 11 shows the correlation between these chosen features and the others for module 3. Except for E1, E2, D2, and D3, most features are highly correlated with at least one chosen feature. This suggests that the feature set we selected is composed of the least number of features containing a majority of the useful information for the classification task, but that some features could be replaced by one or more of those not chosen. This in turn may provide additional flexibility for screening, for example, in instances where a particular behavior is not exhibited by the child during a video clip or short observation session.

**Table 11:** Correlations between used features and not used non-indicator features for module 3

Feature	A2	A4	A8	B3_miss	B2	B7	B8	D4	D3_miss	Male
B10	0.641	0.270	0.422	− 0.07	0.425	0.172	0.554	0.228	− 0.07	0.023
B11	0.487	0.195	0.317	− 0.06	0.324	0.171	0.521	0.362	− 0.06	0.061
A1	0.387	0.293	0.328	− 0.03	0.189	0.163	0.343	0.037	− 0.00	− 0.03
Age_months	0.308	0.119	0.085	0.006	0.257	− 0.01	0.334	0.095	− 0.00	0.024
A3	0.335	0.252	0.167	− 0.09	0.321	0.087	0.361	0.273	− 0.09	0.048
A5	0.231	0.285	0.173	− 0.07	0.256	0.071	0.341	0.294	− 0.08	0.023
A7	0.366	0.086	0.274	− 0.05	0.222	0.122	0.320	0.052	− 0.06	0.025
A6	0.481	0.346	0.385	− 0.12	0.338	0.165	0.495	0.246	− 0.14	0.054
C2	0.365	0.184	0.408	− 0.06	0.250	0.157	0.388	0.168	− 0.05	0.126
C1	0.347	0.147	0.370	− 0.03	0.271	0.125	0.369	0.127	− 0.04	0.099
E1	0.133	0.076	0.171	− 0.03	0.103	0.133	0.149	0.155	− 0.03	0.059
E3	0.029	0.100	0.075	− 0.02	0.101	0.033	0.062	0.068	− 0.02	0.001
E2	0.254	0.078	0.230	− 0.02	0.122	0.108	0.218	0.243	− 0.03	0.031
B9	0.455	0.271	0.329	− 0.09	0.381	0.188	0.518	0.337	− 0.08	0.041
B4	0.180	0.113	0.138	− 0.02	0.116	0.170	0.170	0.139	− 0.02	0.011
B5	0.476	0.147	0.255	− 0.07	0.345	0.105	0.406	0.079	− 0.06	0.033
B6	0.369	0.101	0.267	− 0.07	0.237	0.087	0.287	0.125	− 0.06	0.023
B1	0.261	0.141	0.141	− 0.11	0.283	0.064	0.278	0.221	− 0.09	0.057
B3	0.507	0.171	0.320	− 0.04	0.480	0.090	0.502	0.184	− 0.04	0.017
D2	0.059	0.148	0.075	− 0.04	0.108	0.092	0.087	0.180	− 0.03	0.026
D3	0.105	0.051	0.117	− 0.00	0.044	− 0.00	0.140	0.073	− 0.00	0.039
D1	0.261	0.216	0.171	− 0.06	0.215	0.020	0.255	0.312	− 0.04	0.116

This shows the high correlations between features and so, the need for a robust feature selection technique and classification

We hypothesized that including a binary encoding of whether the data is missing, instead of coding the missing values as a mean of the data or as other values in the same features as existing data, would be useful in this classification task. That a data point is missing might be informative (e.g., it might, in some circumstances, indicate some behavioral trait that led to the item not to be filled out). Given the use of fairly simple (e.g., linear) classifiers, the coding of missing data within a feature that otherwise represents the severity of some trait is undesirable. We found that, in the case of module 3, missing features ranked among the most informative of the top 10 features for classification.

Although somewhat counterintuitive, we found ASD classes no more difficult to classify than classic autism cases and, in some cases, error rates were higher for classic autism cases. This could be due to the particular feature set we extracted. It is possible that the difficulty to label cases is not correlated to severity but type of autism. Our models were selected for performance on the task of separating ASD and classic autism from typical development without distinguishing the former two. While it might be the case that it is easier to distinguish classic autism from controls than it is to distinguish ASD from controls, models that perform as such on both tasks simultaneously may require more complexity and, with that, likely larger amounts of data to distinguish them. Supporting this, we found that a regression task optimized to assign 0 to neurotypical, 1 to ASD (not classic autism), and 2 to classic autism sacrificed accuracy as a two-class (0 vs 1 or 2) thresholding.

The differences in performance for modules 2 and 3 are perhaps due to the numbers of controls, with module 3 having four times more controls than module 2. The low area under the PR curve (when controls are considered positive) seems to support this hypothesis. Good scores for other metrics could also be due to significant age difference between ASD and non-ASD in module 2. The use of reduced feature set of sizes 5 and 10 yielded similar performance. The model with 5 features was simpler and therefore limits overfitting, but a true test of generalization power requires further data collection efforts.

Despite strong evidence for the significant role genetics play in autism risk, there are still no molecular methods for diagnosing ASD. Until reliable biomarkers for autism are identified, behavioral evaluation will necessarily remain the standard for autism diagnosis. However, as the incidence of ASD continues to increase, more and more strain will be placed on diagnostic centers, which often do not have the resources to meet the demand for evaluation of children at risk. This can translate to long wait times for appointments and missed windows of opportunity for beneficial early interventions.

The ADOS [5] has long been found useful, both as a way to gather qualitative observations with which clinicians can make an informed diagnosis and as a standalone scoring mechanism. However, ADOS can present clinical challenges; the full ADOS measure can be time-consuming to apply, and as found in [17], its standard scoring mechanisms can have low specificity, in particular in cohorts with several developmental issues and with cases that are on the spectrum but do not qualify for a classical autism diagnosis.

As a step towards reducing waiting lists at diagnostic clinics, strides have been made to develop mobile screening systems for risk of ASD and other related disorders [9–13]. If such a mobile system could accurately detect ASD from children at risk for developmental delays in general, it would provide utility both for triaging patients in need of more formal clinical evaluations as well as for providing feedback to parents during the often long and arduous process of diagnosis. The classification experiments performed here supports the claim that accurate ASD detection can be performed using the responses to a small set of behavioral features. Previously, we have shown that the behavioral features captured in the ADOS evaluation can be measured in short, unstructured home videos [18]. Considering this result, the classification system described here has potential for utility in shorter format approaches potentially including video-based home screening using mobile devices.

### Conclusions & limitations

This study was limited by the contents of available data sets. The phenotype data used here were obtained from publicly available autism research data repositories, which have relatively few ADOS examinations for non-ASD control subjects. However, it is important to note that the control subjects who did receive an ADOS examination were initially suspected of having autism and later failed to meet the cutoffs for formal diagnosis. Therefore, although the number of controls available for model training was minimal, the controls used may represent challenging “edge” cases that help the classifier create a robust boundary between actual ASD cases and cases that exhibit some ASD-like characteristics but who may have another underlying condition. Considering that ASD-specific screening using ADOS is most often performed for children that are suspected of having ASD, the high accuracy of our classifier on this control set is a good representation of its performance in the actual population that requires ASD screening. Of course, more training data points would improve the overall accuracy of the classification system. We plan to conduct future studies to tune the classifiers as more control data become available.

The data for modules 2 and 3 contained balance concerns. Namely, in module 2, age and gender were not well balanced between ASD and non-ASD, and in the module 3 data, gender was not well balanced. Gender appeared as the lowest of the top 10 ranked features from the module 3 analysis, suggesting a limited role in classification. In our module 2 analysis, neither gender nor age appeared in our top 5 ranked feature set. While the low ranking of these imbalanced features provides some confidence that they do not negatively impact classification, the possibility remains that the classifier could be capturing correlations with these features, and thus could be performing in a way that would not generalize to the full population. To understand this potential limitation better, we retrained with age and gender features removed and achieved comparable results. We also computed correlation matrices (Tables 7 and 10) of selected features and age and gender and found correlation of age and gender with selected features to be negligible. While these results suggest that the imbalance within these features did not have a biasing effect, data collected with better balance will be an important next step to determine the generalization of our classifiers.

## Abbreviations

ADOS: Autism diagnostic observation schedule
ADIR: Autism diagnostic interview revised
ASD: Autism spectrum disorder
